# Mindfulness-Based Strategies for Improving Sleep in People with Psychiatric Disorders

**DOI:** 10.1007/s11920-022-01370-z

**Published:** 2022-10-13

**Authors:** Allie L. Peters, William J. Saunders, Melinda L. Jackson

**Affiliations:** 1 School of Psychological Sciences, Turner Institute for Brain and Mental Health, Monash University, 18 Innovation Walk, Clayton, Victoria Australia; 2 Danny Frawley Centre, Moorabbin, Victoria Australia

**Keywords:** Insomnia, Meditation, Treatment, Depression, Anxiety, Post-traumatic stress disorder

## Abstract

***Purpose of the Review*:**

To review the recent literature on mindfulness-based strategies for improving self-report and objective measures of sleep, in individuals with psychiatric disorders.

***Recent Findings*:**

Currently, research provides some support for the use of mindfulness-based interventions to improve sleep amongst individuals with psychiatric comorbidities. The strongest evidence was for the use of standardized programs, particularly for improving sleep in anxiety and depressive disorders. There is a paucity of well-controlled studies using validated subjective or objective measures of sleep. As these interventions were not specifically designed to target sleep, observed improvements may be an indirect consequence of reduced psychiatric symptoms.

***Summary*:**

There is insufficient research into the application of mindfulness-based strategies to improve sleep or treat sleep disorders in people with psychiatric disorders. Well-controlled studies using standardized, mindfulness-based interventions developed to target sleep, such as mindfulness-based therapy for insomnia, may optimize the potential benefits of mindfulness for sleep in psychiatric populations.

**Supplementary Information:**

The online version contains supplementary material available at 10.1007/s11920-022-01370-z.

## Introduction

Psychiatric disorders are highly prevalent, affecting 1 billion people globally [1], with major depression contributing the second largest global burden of disease [2]. Sleep disturbance is common across the spectrum of psychiatric disorders, with between 50 to 80% of psychiatric patients reporting disturbed sleep. Indeed, the Diagnostic and Statistical Manual of Mental Disorders (DSM-V) criteria for most psychiatric conditions includes sleep disturbance and/or hypersomnolence.

It is also well recognized that there is a strong relationship between insomnia and psychiatric disorders. Insomnia is a highly prevalent sleep disorder [3], characterized by sleep disturbances at least three times a week, including difficulty falling or staying asleep or waking too early, despite adequate opportunity to sleep, and accompanied by significant daytime impairment or distress [4]. It is thought that insomnia and psychiatric conditions not only share common underlying causes, but may also have a complex, bidirectional relationship [5]. For example, as well as sleep disturbance being a common presentation in psychiatric disorders, between 40 and 60% of individuals with insomnia suffer from a concomitant mental disorder, most commonly depression and anxiety [6]. There have also been relationships observed between insomnia and impulsivity [7, 8], substance misuse disorders [9–11], bipolar disorder [12], obsessive–compulsive disorder [13], paranoia [14] and schizophrenia [15]. Insomnia can influence the onset and trajectory of psychiatric conditions, with the presence, severity and chronicity of insomnia found to be predictors of psychiatric illness [6, 16]. For example, insomnia is a significant risk factor for the development of a first episode of depression [17], for recurrent depression [18, 19], and is a significant predictor for subsequent depressive episodes [20]. Concerningly, individuals experiencing psychiatric illness with comorbid sleep disorders fare worse with regard to quality of life and disease severity than individuals without sleep problems [21]. Sleep problems in individuals with depression have been noted as significant predictors of suicidal ideation and suicidal behaviour [22]. Additionally, individuals with comorbid anxiety and insomnia have poorer reported quality of life than those with anxiety only [23]. Thus, it is important to consider sleep in the context of assessment and treatment of psychiatric disorders.

One mechanism which may underpin this relationship between sleep and mental health is hyperarousal. This is commonly experienced by people with a wide variety of mental illnesses, such as anxiety disorders, post-traumatic stress disorder (PTSD), attention-deficit hyperactivity disorder (ADHD) and obsessive–compulsive disorder, and is a criterion of these disorders in the DSM-V [4]. Hyperarousal has been conceptualized to include cognitive, somatic and cortical overactivation, and has the potential to profoundly impact both the quality and quantity of sleep [24]. Ong et al. [25] provide an explanation and a model of cognitive arousal with respect to sleep [25]. As such, a treatment that addresses hyperarousal may be of additional value to those with comorbid insomnia and mental illness.

There is some evidence that treatment of anxiety through psychotherapy and cognitive behavioural therapy (CBT) can have beneficial effects on sleep [26]. Access to effective treatment can be problematic for some people, especially if they are unaware of the range of available therapies, find them difficult to access or ineffective, or if they do not wish to be labelled as having a mental illness [27–29]. Additionally, people with affective symptoms (e.g., anxiety, depression) are also more likely to use complementary and alternative therapies than mainstream and primary care treatments [30]. Thus, a broader range of new and effective treatment strategies, including complementary and alternative therapies, is required to meet this growing demand.

Mindfulness training is a core component of a ‘third wave’ of psychotherapies that are effective in treating a range of psychiatric disorders [31]. Mindfulness is the self-regulation of attention, which involves sustained attention, attention switching and the inhibition of elaborative processing [32]. Ong and Moore [33] posit that mindfulness may improve sleep via reductions of both primary and secondary arousal. Primary arousal is the mental activity directly related to the inability to sleep, such as beliefs about daytime consequences. Secondary arousal is the relationship with thoughts about sleep such as the tendency to create bias in the attention and perception of sleep related thoughts [33]. Mindfulness principals are proposed to treat insomnia via three related mechanisms: [1] increasing awareness of the mental and physical states that arise when experiencing insomnia symptoms, [2] shifting mental processes to reduce sleep-related arousal and [3] promoting a mindful stance to respond when symptoms of insomnia arise [34]. If mindfulness can improve primary and secondary arousal, it may provide a valuable treatment option, particularly for those with mental illness. Mindfulness-based cognitive therapy (MBCT) was originally developed to treat chronic depression [35], and is shown to be efficacious in reducing depressive symptoms [36, 37]. The purpose of these therapies is to normalize one’s thinking patterns so that mild sadness does not escalate into a more severe state [38].

There is also some evidence that MBCT and other mindfulness-based interventions (MBI) may be effective at reducing sleep problems in individuals with comorbid psychiatric disorders. A recent systematic review and meta-analysis focused on identifying and evaluating the clinical importance of different MBI programs on sleep, focusing on individuals with anxiety and depression only [39]. This review evaluated 10 randomized control trials (RCT) of interventions including mindfulness-based stress reduction (MBSR) and MBCT, as well as less traditional approaches such as mindfulness meditation, internet mindfulness meditation intervention (IMMI) [40], mindfulness-based touch therapy (MBTT) [41] and mind–body bridging [42]. They reported the largest effect sizes for MBTT, followed by MBCT, with smaller effects for IMMI and MBSR. Significant effects on sleep problem improvement are shown in all reviewed MBI programs, except mindfulness meditation approaches, for which the effect size were small [39]. To extend the current available reviews, the current study provides an overview of MBIs across a range of psychiatric illnesses and includes uncontrolled studies, as we believe that at this stage of research in the area, they are still important to consider. Therefore, the aim of this review is to summarize the evidence for the use of MBI’s as an intervention to improve both objective and subjective measures of sleep in individuals with psychiatric disorders.

## Results

An extensive literature search was completed using the following databases (Google Scholar, Ovid MEDLINE(R), Scopus (Elsevier), PubMed), and the following search terms: (Mindfulness, psychiatric populations, intervention, anxiety, anxiety disorder, depress*, depression, depressive disorder, schizophrenia, OCD, PTSD, bipolar disorder, ADHD, personality disorder). Studies were included if a mindfulness-based intervention was delivered to a sample of individuals with psychiatric disorders and a sleep outcome measure was used. Table 1 summarizes the selected studies.

**Table 1:** Summary of studies examining the effect of mindfulness-based interventions (MBIs) on sleep in psychiatric disorders

Disorder	Reference	Sample (age, sex)	*N* (group size)	Intervention (components)	Duration	Sleep outcome measures	Summary of findings
**ADHD**							
	Fried et al. (2022) [45]	Children with ADHD (6–12 years, *M* (*SD*) = 9.2 (1.6) years, 78% male)	18	Age-appropriate, brief, guided digital mindfulness meditation exercises (Headspace)	4 weeks, 1–10 min of meditation (at least ≥ 1 min)	Children’s Sleep Habit Questionnaire (parent-report measure)	Significant improvements in sleep problems (d = .47 [− .02, .96], *p* = .04) after 4 weeks
	Zaccari et al. (2021) [47]	Children with ADHD (7–11 years, *M* (*SD*) = 8.94 (1.25) years, 72% male)	25 (MOM = 15, active control = 10)	Mindfulness-oriented meditation (mindfulness of breath, body parts and thoughts; presented as games)	8 weeks, 3 group sessions per week, 6–30 min (increasing per week). At-home practice encouraged	Actigraphy, Sleep Disturbance Scale for Children (parent reported)	Significant improvement in parent-observed initiation and maintenance of sleep in the MOM group but not in the control group (*p* = 0.002). Sleep difficulties reduced to within the normal range in the MOM group. No group difference in actigraphy outcomes
	van de Weijer-Bergsma et al. (2012) [46]	Adolescents with ADHD (11–15 years, *M* = 13.4 years, 50% female)	10 (including 19 parents and 7 teachers)	Mindfulness training and mindful parenting training	8 weeks, 1 group session per week, 1.5 h. Booster session 8 weeks post-intervention	Flinders Fatigue Scale	No significant change in adolescent fatigue or mindfulness (both parent- and self-reported)
**Anxiety**							
	Horenstein et al. (2019) [49]	Adults with Social Anxiety Disorder (≥ 18 years*,* M* (SD) = 32.70 (7.99) years, 55.6% female)	108 (MBSR = 36, CBGT = 36, wait-list control = 36)	MBSR (modified; 4 extra sessions instead of one-day meditation retreat)	12 weeks, one 2.5-h group session per week	PSQI	MBSR significantly improved sleep quality relative to waitlist (*p* < .01, *η*^2^_p_ = .23), but did not differ from CBGT. MBSR and CBGT significantly reduced social anxiety. This association was not moderated by baseline sleep quality
	Boettcher et al. (2014) [51]	Adults diagnosed with a DSM-IV anxiety disorder (≥ 18 years*, *M* (SD) = 37 (8.9) years, 71% female)	91 (mindfulness = 45, active control (online discussion forum) = 46)	Brief audio-guided mindfulness-based meditations (sitting meditation, mindful movement, body scan, breathing anchor)	8 weeks, 8 modules via website (unguided), twice a day (20 min) for 6 days per week	ISI	Significant pre-to-post-test reductions in insomnia symptoms between groups (*d* = 0.36, *p* = .016) Post-treatment reductions in insomnia remained stable at 6-month follow-up
	Hoge et al. (2013) [50]	Adults with Generalized Anxiety Disorder (≥ 18 years*, *M* (SD) = 39 (13) years, 51% female)	89 (MBSR = 48, stress management control = 41)	MBSR	8 weeks, one 2-h group session per week, plus 1 all-day silent retreat	PSQI	Significant improvements in sleep quality following MBSR compared to control (*p* = .035)
	Vøllestad et al. (2011) [52]	Adults diagnosed with a DSM-IV anxiety disorder (18–65 years*, *M* (SD) = 42.5 (11.3) years, 51% female)	76 (MBSR = 39, wait-list control = 37)	MBSR	8 weeks, one 2.5-h group session per week, plus half-day meditation retreat. Daily at-home practice	Bergen Insomnia Scale	No significant differences in insomnia symptoms between groups post-treatment Significant decrease in individuals meeting diagnostic criteria for insomnia after MBSR compared to controls (*p* = .003)
	Yook et al. (2008) [53]	Adults diagnosed with a DSM-IV anxiety disorder (31–51 years, *M* (SD) = 41.1 (6.3) years, 58% male)	19	MBCT	8 weeks, one 2-h group session per week	PSQI	Post-treatment global PSQI scores were significantly lower than baseline global PSQI scores (Z = − 3.46, *p* < .001, *d* = 1.32) PSQI components: sleep quality (Z = − 2.52, *p* = .01), habitual sleep efficiency (Z = − 3.00, *p* < .001), sleep latency (Z = − 2.33, *p* = .02), use of sleep medications (Z = − 2.11, *p* = .04) and daytime dysfunction (Z = − 2.49, *p* = .01) improved after MBCT. No change in sleep duration
**Mixed anxiety/depression**							
	Lavin et al. (2021) (60)	Adults undergoing haemodialysis treatment with clinically significant self-reported anxiety and/or depression (*M* (SD) = 68.7 (6.4) years, 53% male)	19 (mindfulness = 12, active control (health enhancement) = 7)	Brief mindfulness intervention (including guided mindfulness meditations from MBCT)	8 weeks, 2 chair-side sessions per week, 20 min each. 10 min daily at-home mindfulness practice	Athens Insomnia Scale 8, Actigraphy (consolidation of daily inactivity)	No significant differences in insomnia or actigraphy outcomes between groups. Both groups demonstrated clinically important reductions in self-reported insomnia at follow-up Reductions in insomnia symptoms were associated with improvements in depression scores
	Chan et al. (2020) [61]	Adults diagnosed with a DSM-IV anxiety and/or depressive disorder (18–70 years*, *M* (*SD*) = 50.5 (10.9) years, 70.6% female)	187 (MBCT = 62, HQCT = 62, wait-list control = 63)	MBCT	8 weeks, one 2-h group session per week	PSQI	MBCT group significantly improved sleep quality compared to controls (*d* = 0.25 at post- and 0.56 at 8 weeks post-intervention)
	Nissen et al. (2020) [62]	Adult cancer survivors screening positive for anxiety and/or depressive symptoms (≥ 18 years, *M* = 55.45, 91% female)	150 (iMBCT = 104, TAU = 46)	iMBCT (adapted for cancer survivors)	8 weeks, 1 module per week, weekly training diary and therapist feedback	ISI	No significant differences in insomnia severity between groups at 10 weeks or 6 months post-treatment (*d* = 0.04–0.06)
	Zhang et al. (2017) [63]	Adults with leukaemia and clinically significant self-reported anxiety and/or depression (17–71 years, *M* (*SD*) = 38.35 (8.93) years, 60% male)	65 (MBSR = 33, TAU = 32)	MBSR (modified; mindfulness-based psychological care)	5 weeks, 1 individual session per week, 30–40-min audio-guided meditation during IV infusion	PSQI	Following MBSR, significantly improved sleep quality (global PSQI) compared to baseline (*p* = .002) and controls (*p* < .001)
	Pinniger et al. (2013) [64]	Adults with moderate to severe self-reported stress, anxiety or depression (18–68 years, *M* = 39.5 years, 89.1% female)	64 (meditation = 11; tango = 18; exercise = 12, control = 23)	Mindfulness meditation (breathing exercises, mindful eating, body scan, walking meditation, music meditation)	8 weeks, 1 group session per week, 1.5 h	ISI, Fatigue Severity Scale	Significant decrease in insomnia scores for the tango group (*p* < .05, *d* = .71). Improvement in meditation group insomnia scores from pre-to-post-treatment, and to follow-up; however, this was not significant. Fatigue severity scores did not significantly differ between groups
**Depression**							
	Schramm et al. (2016) [54]	Adults diagnosed with a DSM-IV depressive disorder (18–75 years*, *M* (*SD*) = 48.20 (9.28) years, 64% female)	25 (MBCT = 9, CBASP = 8, TAU = 8)	MBCT	8 weeks, one 2.5-h group session per week. 40-min homework practice per day	Cardiopulmonary coupling and ECG-derived sleep outcomes (TST, wake, SE)	CBASP group had significantly less wake compared to MBCT (*p* = 0.039) and TAU (*p* = .004) at post-treatment
	Stötter et al. (2013) [41]	Adults diagnosed with ICD-10 moderate depression (*M* (*SD*) = 42.1 (10.6) years, 78.57% female)	28 (MBTT = 14, TAU control = 14)	Mindfulness-Based Touch Therapy	10 individual sessions over 8 weeks, at-home mindful breathing practice and body scan exercises	Hamilton Depression Rating Scale (insomnia subscales)	Compared to controls, sleep maintenance insomnia was significantly reduced after MBTT (*p* = .003). 64% of the MBTT group reported an improved ability to remain asleep. 43% of MBTT group reported improvements in sleep onset insomnia compared to 7% of controls; however, this was not significant (*p* = .114)
	Wahbeh (2018) [40]	Older adults with self-reported clinically significant depression (55–80 years*, *M* (*SD*) = 64.8 (6.2) years, 80% female	50 (IMMI = 26, wait-list control = 24)	Internet Mindfulness Meditation Intervention	6 weeks, 1 individual session per week, 1 h. 20 min of at-home practice	ISI	Compared to controls, insomnia severity in the IMMI group significantly improved post-intervention (*p* < .001) Reductions in insomnia severity in IMMI group were sustained at 7 weeks (*p* < .001)
	Britton et al. (2012) [56]	Adults with partially remitted DSM-IV depression using antidepressants (24–61 years, *M* (*SD*) = 47.0 (10.5) 80.76% female)	24 (MBCT = 15, controls = 11)	MBCT	8 weeks, one 3-h group session per week. 1 all-day silent retreat. At-home audio-guided practice	Polysomnography (SWS, TIB, TST, SE, SOL, WASO, TWT, stage 1 sleep, SWS) and sleep diaries	For objective sleep, greater decrease in TWT for the MBCT group relative to controls (*p* = .035, *η*_p_^2^ = .20) For subjective sleep, greater decrease in TWT (*p* = 0.046, *η*_p_^2^ = .19) and greater increase in SE (*p* = 0.007, *η*_p_^2^ = 0.33) for the MBCT group compared to controls
	Britton et al. (2010) [55]	Adults with partially remitted DSM-IV depression not using antidepressants (33–64 years, *M* (*SD*) = 47.7 (7.6), 76.9% female	26 (MBCT = 14, control = 12)	MBCT	8 weeks, one 3-h group session per week. 1 all-day silent retreat during week 6. At-home audio-guided practice, assigned as homework	Polysomnography (SWS, TIB, TST, SE, SOL, WASO, TWT, stage 1 sleep, SWS, number of awakenings) and sleep diaries	Increased arousal (increased awakenings, stage 1 sleep) and a significant decrease in SWS following MBCT, relative to controls. No significant effects for all other PSG measures Improvements in subjectively reported sleep after MBCT (SE: *p* < .001, *η*_p_^2^ = .49; TST: *p* = .006, *η*_p_^2^ = .35; WASO: *p* = .006, *η*_p_^2^ = .35; number of awakenings: *p* = .006, *η*_p_^2^ = .34; SOL: *p* = .002), but not over and above the control group
**Heterogeneous psychiatric diagnoses**							
	Moussaoui et al. (2021) [68]	Adults with heterogeneous psychiatric diagnoses (≥ 18 years*, *M* (*SD*) = 39.47(15.67) years, 55% male)	20 (mindfulness = 11, control = 9)	Mindfulness-oriented intervention (Tai Chi, yoga-inspired gentle stretching, walking meditation)	One 10-min group session per day over 5 days	Athens Insomnia Scale	No significant group differences in insomnia severity post-treatment (*p* = 0.09)
	Foulk et al. (2014) [43]	Older adults diagnosed with heterogeneous psychiatric disorders (61–89 years, *M* = 72.9, 64% female	50	MBCT (adapted for older adults)	8 weeks, one 2-h group session per week, half-day silent retreat	Sleep Preoccupation Scale	Significantly reduced frequency of sleep problems (*p* = 0.04); mean symptom improvement of 14.5%
	Biegel et al. (2009) [70]	Adolescents with heterogeneous DSM-IV psychiatric diagnoses (14–18 years, *M* (*SD*) = 15.35 (1.20), 73.53% female)	102 (MBSR = 50, TAU = 52)	MBSR	8 weeks, one 2-h group session per week, 20–35 min of at-home practice	Single item self-report measure of sleep quality (Likert scale)	MBSR + TAU significantly improved sleep quality compared with TAU (*p* = .02); however, this was due to a significant deterioration in self-reported sleep quality within the TAU group, rather than improved sleep within the MBSR group
	Ree and Craigie (2007) [28]	Adults with heterogeneous DSM-IV psychiatric diagnoses (18–73 years, *M* (*SD*) = 39.5 (15.27) years, 77% female)	26	MBCT	8 weeks, 2.5-h group session per week, 45 min at-home practice. 2-h follow-up group session at 3 months post-treatment	ISI	ISI scores significantly reduced post-treatment (intention-to-treat: *p* < .01, *d* = .78), and maintained at 3 month (intention-to-treat: *p* < .01, *d* = .79) ISI scores were in the normal range post-treatment (intention-to-treat: *M* = 6.18, *SD* = 5.09) and follow-up (Intention-to-treat: *M* = 6.14, *SD* = 5.09), with large effects sizes
**PTSD**							
	Wahbeh et al. (2016) [65]	Military veterans diagnosed with DSM-IV PTSD (25–65 years*, *M* = 52.12 (12.43), 94% male)	102 (MM = 27, SB = 25, MM + SB = 25, control = 25)	Body scan mindfulness meditation and/or slow breathing with biofeedback	6 weeks, one 20-min individual session per week, at-home practice for 20 min each day	PSQI	Sleep quality in the MM group differed from SB post-treatment (*p* = .009). Significant improvement in sleep quality in the MM group; however, scores remained above the threshold (> 5) for significant sleep disturbance
	Nakamura et al. (2011) [42]	Military veterans with self-reported sleep disturbance and clinically significant PTSD symptoms (18–70 years*, *M* = 52.05 (10.35) years, 95% male)	58 (MBB = 33, active control = 25)	Mind–body bridging	3 weeks, 2 sessions total, 1 per week. At-home practice encouraged, particularly at bedtime	Medical Outcomes Sleep Study Survey	Sleep-focused MBB in two sessions reduced self-reported sleep disturbance compared to controls (*p* = .006) Sleep disturbance in the MBB group was lower at week 1 (*p* = .021) and post-treatment (*p* < .001) compared to controls. Significant within-group decreases in sleep disturbance after MMB at week 1 (*p* < .001) and post-treatment (*p* < .001)

*ADHD *attention-deficit hyperactivity disorder, *CBASP *cognitive behavioural analysis system of psychotherapy, *CBGT *Cognitive Behavioural Group Therapy, *ECG *electrocardiogram, *iMBCT *internet MBCT, *IMM* internet mindfulness meditation intervention, *ISI *Insomnia Severity Index, *MBB *mind–body bridging, *MBCT *mindfulness-based cognitive therapy, *MBSR *mindfulness-based stress reduction, *MBTT *mindfulness-based touch therapy, *MM *mindfulness meditation, *MOM *mindfulness-oriented meditation, *PSG *polysomnography, *PSQI *Pittsburgh Sleep Quality Index, *PTSD *post-traumatic stress disorder, *SB *slow breathing, *SE *sleep efficiency, *SOL *sleep onset latency, *SWS *slow-wave sleep, *TAU *treatment as usual, *TIB *time in bed, *TST *total sleep time, *TWT *total wake time, *WASO *Wake After Sleep Onset

*inclusion criteria, not actual range

**Fig. 1:**
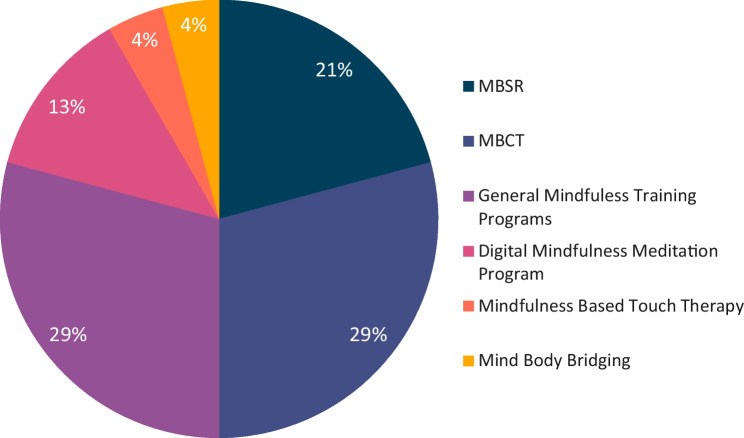
Range and frequency of mindfulness-based interventions delivered studies of sleep in psychiatric disorders observed in the current review

As shown in Fig. 1, MBCT, MBSR and General Mindfulness Training programs had the highest representation with respect to types of MBI’s used. Although manualized MBCT and MBSR programs were the most frequently used MBIs, these interventions were often adapted to suit specific populations of people with mental health disorders, for example one study adapted the manualized MBCT program to suit older adults by removing the yoga component [43]. There is high variability in the category ‘General Mindfulness Training Programs’, though many of these interventions involve meditations and exercises used in MBCT and MBSR such as sitting meditation, body scan, mindful movement and mindful eating. We also found less traditional delivery methods of mindfulness, for example mind–body bridging [42] and MBTT [41]. An overview of the findings for each intervention, categorised by diagnosis of participants, can be found in Table 2.

**Table 2:** Summary of outcomes of studies of the effect of MBIs on sleep, based on intervention type and population

	MBSR	MBCT	General mindfulness meditation/training	Digital mindfulness program	Mindfulness-based touch therapy	Mind–body bridging
ADHD—child	-	-	✓	✓	-	-
ADHD—adolescent	-	-	$\times$	-	-	-
Anxiety	✓✓✓	✓	✓	-	-	-
Mixed anxiety/depression	✓	✓	$\times\times$	$\times$	-	-
Depression	-	✓$\times\times$	-	✓	✓	-
Mixed sample	-	✓✓	$\times$	-	-	-
Mixed sample—adolescent	$\times$	-	-	-	-	-
PTSD	-	-	✓	-	-	✓

*MBSR *mindfulness-based stress reduction, *MBCT *mindfulness-based cognitive therapy, *ADHD *attention-deficit hyperactivity disorder, *PTSD *post-traumatic stress disorder

NB: $\times$ null result for sleep, ✓ positive result for sleep

### ADHD

Sleep problems are reported in an estimated 25–50% of individuals who have ADHD, and adults who do not get the recommended sleep duration are more likely to report ADHD symptoms [44]. Three studies have investigated the use of MBI’s in sleep in people with ADHD [45–47]. One study investigated a brief mindfulness training intervention for adolescents in addition to mindful parenting training [46] and two studies with children [45, 47]; no studies with adult ADHD participants were found. Zaccari et al. [47] investigated the impact of an 8-week mindfulness-orientated meditation involving three meditation exercises based on MBSR as ‘games’ compared with an active control condition involving emotional awareness and recognition in children with ADHD. This study yielded significant improvements in parent-observed sleep in the mindfulness-orientated meditation group compared to the control group, but no group differences were observed for objective sleep (actigraphy) [47]. Another study of children with ADHD implemented a 4-week digital meditation application, observing significant improvements in parent-reported sleep difficulties and anxiety [45]. No significant changes were found in fatigue or mindfulness following an 8-week mindfulness-training program in adolescents [46].

### Anxiety Disorders

Sleep disturbances are seen in approximately 70% of individuals with anxiety disorders [48]. It is most common for anxiety disorders to occur simultaneously with or precede the onset of insomnia, but insomnia can also occur prior to the onset of anxiety [6].

The effectiveness of MBIs has been examined across a number of anxiety disorders, including social anxiety disorder [49], generalized anxiety disorder [50] and heterogeneous anxiety disorders [51–53]. Three studies used traditional face-to-face MBSR [49, 50, 52] and one used MBCT [53], with one study examining the effectiveness of used brief, digital audio-guided meditations [51]. Results from the MBSR studies are mixed, with two studies demonstrating significant improvements in sleep quality using the Pittsburgh Sleep Quality Index (PSQI) relative to controls in individuals with Generalized Anxiety Disorder and Social Anxiety Disorder [49, 50], whereas Vøllestad et al. [52] found no significant difference in insomnia symptoms measured by the Bergen Insomnia Scale after MBSR compared to wait-list [52].

There is also evidence that self-directed internet-delivered meditation practice may have some benefits for this population, with moderate effect sizes observed in insomnia severity between active and control groups at 8-week follow-up (*d* = 0.45 (0.14–0.76), which were sustained at 6-month follow-up [51]. No objective measure of sleep was reported in any of these studies. This represents the strongest body of research supporting MBIs for sleep for a particular psychiatric disorder.

### Depression

Two studies investigated MBIs for sleep in samples with depressive disorders [41, 54]. Schramm et al. [54] compared MBCT, cognitive behavioural analysis system of psychotherapy and treatment as usual in a small sample of chronically depressed patients [54]. They examined sleep objectively using cardiopulmonary coupling analysis. Results indicated the psychotherapy group had more stable sleep and less wake compared with controls and less wake time compared to the MBCT group at follow-up. While this is one of the few studies to use objective measures of sleep, the study was limited by the small sample size and no self-report measures of sleep quality.

Stötter et al. [43] implemented an RCT of mindfulness-based touch therapy (MBTT) compared to treatment as usual in 28 patients with moderate recurrent episodic depression [41]. MBTT is an experientially, body-oriented form of psychotherapy, involving the use of touch experiments while the client is in a state of inner mindfulness in order to facilitate access to the unconscious. This therapy works from the opposite end of the information processing hierarchy than traditional meditation, resulting in the integration of immediate bodily experience with mindful cognitive self-awareness [41]. This study yielded the largest effect size as reported in recent review and meta-analysis [39], with 64% of patients reporting improvements in symptoms of sleep maintenance insomnia.

There is growing evidence for the use of MBIs for sleep in individuals with subclinical depression [40, 55, 56]. Significant improvements in insomnia symptoms were observed in one RCT using an internet-based mindfulness program compared to wait-list in older adults with mild-moderate symptoms of depression [40]. Britton et al. [56] examined the impact of MBCT on polysomnographic and subjective sleep in a sample of adults with partially remitted depression [55] and antidepressant medication users with residual sleep complaints [56]. In their first study, increased arousal in the MBCT group was observed, including increased awakenings and stage one sleep, and a significant decrease in slow wave sleep relative to controls, contrary to expectations [55]. The amount of mindfulness practice was correlated with increased polysomnography arousals, awakenings and stage one sleep in a dose-dependent fashion. Britton also reported within-group improvements in subjectively reported sleep in the MBCT group, but this did not differ from the control group at follow-up. These results are not consistent with the preposition that mindfulness meditation has the potential to decrease arousal [25, 57, 58] and inconsistent other studies that have found significant reductions in hyperarousal following mindfulness-based therapy for insomnia (MBT-I) (e.g., [59]). In a follow-up study of current antidepressant users with residual sleep complaints, significant reductions in objective and subjective total wake time and improvements in sleep efficiency were observed, but no change in sleep depth [56].

### Mixed Anxiety/Depression in Patients with Comorbidities

Five studies investigated MBIs for sleep in mixed anxiety/depression groups [60–64]. Most of these studies involved participants with complex comorbidities, including leukaemia [63], cancer survivors [62] and individuals undergoing haemodialysis [60].

A large RCT of cancer survivors observed no significant changes to insomnia severity on the Insomnia Severity Index (ISI) following a 10-week digital MBCT program [62]. The average ISI scores were in the sub-clinical range, and there was significant non-adherence, with only 46 of 104 participants randomized to the intervention group completing all treatment modules. In contrast, RCTs of individuals from a psychiatric outpatient clinic observed significant improvements in sleep quality and sleep problems after an in-person MBCT program [61]. Significant improvements in sleep quality have also been observed following a modified MBSR program in patients with leukaemia [63].

One study of patients undergoing haemodialysis treatment measure did not find a significant improvement in objective sleep outcomes after a brief mindfulness intervention; however, the treatment group demonstrated clinically significant improvements in insomnia symptoms [60]. Similarly, a study investigating 8 weeks of group meditation training compared to tango dance, exercise and wait-list control in adults with anxiety and/or depression found no difference in insomnia at follow-up [64].

### PTSD

Post-traumatic stress disorder is a serious public health concern, with rates amongst veterans reported to be 3.5% prevalence per year. Nightmares and sleep disturbance are common symptoms of PTSD and form part of the diagnostic criteria. There is growing evidence of the benefits of mindfulness meditation approaches for PTSD symptoms; however, only two RCTs have investigated the effect of MBIs on sleep outcomes in individuals with PTSD, both including veteran samples [42, 65]. One study utilized body scan mindfulness meditation versus three control conditions (slow breathing, sitting quietly and biofeedback) [65]. There was a significant reduction in PSQI scores for the meditation group; however, the average post-treatment score was still in the clinically significant range at 8.4, with scores > 5 being considered as a significant sleep disturbance. The second study utilized a mind–body bridging intervention, which is a short-term mind–body intervention informed by Western psychoanalysis and Eastern Zen-like contemplative practices [42, 66, 67]. Results of this study suggested that sleep-focused mind–body bridging greatly reduced patient-reported sleep disturbance compared to a control group after two sessions [42]. These studies provide preliminary support for the use of MBIs for addressing sleep issues in PTSD.

### Heterogeneous Samples

A number of studies examined MBIs in a wide-range of DSM-IV disorders beyond affective disorders, including eating disorders, ADHD, substance-related, bipolar, schizoaffective, schizophrenia, psychosis and adjustment disorders [28, 64, 68–70].

Two studies have examined within-group changes in sleep after group MBCT programs. Ree and Cragie [28] examined the effectiveness of an MBCT program in a heterogeneous group of adult outpatients with psychiatric presentations [28]. Significant improvements on all measures, including insomnia severity, were achieved following the MBCT intervention, with maintenance of gains at 3-month follow-up. Mean ISI scores were in the normal range post-treatment and at follow-up, with large effect size [28]. Similarly, Foulk et al. [43] found a significant reduction in sleep problems in a sample of older adults with depression and anxiety disorders after an 8-week MBCT program [43]. In an RCT of 102 adolescents from a psychiatric outpatients clinic, Biegel et al. [70] found that MBSR resulted in significant improvements in a range of outcomes, including a small improvement in sleep quality [70].

The outcomes of studies examining the impact of less structured MBI programs have been less promising. An RCT of adults were given a mindfulness-orientated intervention compared to treatment as usual observed no significant differences between groups on the Athens Insomnia Scale [68].

## Discussion

Mindfulness-based interventions are increasingly utilized in clinical practice for the treatment of a range of psychiatric disorders. This review aimed to summarize the evidence for the use of MBIs as an intervention to improve sleep in individuals with psychiatric disorders. The body of literature presented provides support for the use of MBIs for improving sleep in those with comorbid mental health issues, particularly structured, group format programs MBCT and MBSR. Out of a total of 24 studies reviewed, only 7 studies failed to find a significant improvement in sleep outcomes. All studies investigating MBCT observed favourable outcomes for self-reported sleep across a range of different populations. There was most support for the use of MBSR in anxiety disorders, with three studies finding that MBSR improved sleep in those with anxiety disorders. The current literature, while suggesting promise for the use of MBIs for insomnia in those with mental illness, is very limited, with very few replication studies, indicating a need for further research.

There are a number of considerations when reviewing the current literature. There is a large range of psychiatric disorders examined in this review, with very few having more than two RCTs conducted in the same disorder population. Similarly, participants within and between studies varied considerably with regard to medication use. Many pharmacological treatments used in these disorders may also interact with sleep (e.g., SSRIs, benzodiazepines). Indeed, many patients using pharmacotherapy often turn to MBIs to assist with reducing medication; how effective this approach is would be an interesting avenue for future research.

There was also a large variability in the sleep measures used, with around half of the studies using a validated insomnia measure. There is currently limited evidence for the impact of MBIs on objective measures of sleep, with only 5 studies using actigraphy or polysomnography. Future studies should aim to include both a validated insomnia scale (e.g., ISI) and objective monitoring of sleep to gain a more holistic assessment of sleep changes. Furthermore, there is a need for further evidence of the efficacy of MBIs for sleep in the psychiatric disorders presented in this review, as well as other psychiatric conditions that have not yet been studied. For example, examination of MBIs for individuals with obsessive–compulsive disorder may be beneficial given the physiological and cognitive components of the disorder that are likely to impact on sleep. While there is some research into the effects of MBI in patients with schizophrenia, schizoaffective disorder or bipolar disorder [71] and autism spectrum disorder [72], these studies only used qualitative or non-validated sleep measures. Future research with validated outcome measures in these psychiatric disorders would be beneficial.

Besides the standardized 8-week MBCT and MBSR programs utilized in some studies, there was variability in treatment structure and duration, ranging from 5 days to 12 weeks. Many studies also encouraged participation in between-session mindfulness mediation exercises. The shortest intervention did not demonstrate significant improvement in sleep. Overall, however, there did not seem to be a relationship with dose and response, i.e. there were shorter interventions that revealed a significant improvement in sleep; there were also longer interventions that did not result in a change in sleep outcomes. One factor that may have contributed to these different findings is the amount of home practice participants undertook between sessions. Adherence to ‘homework’ was rarely measured in these studies and may be a factor contributing to null findings. Similarly, programs delivered in group format may be more beneficial for sleep and other outcomes, due to increased adherence and motivation.

The outcomes of these studies are also likely to depend on the severity of sleep problems and insomnia experienced by the patient. To date, most research evaluating MBIs for insomnia has excluded participants with psychiatric conditions to ensure that a ‘pure’ sample of people with insomnia is studied. Although this allows for a clearer conclusion to be made regarding the effectiveness of the treatment from a sleep perspective and is important for the early stages of study into treatment development, such people are not representative of the population of insomnia suffers given the frequency with which mental illness and insomnia co-occur. The mechanisms for how MBIs address sleep are also an interesting question for future research. As the interventions reviewed were not specifically designed to target sleep, observed improvements may be an indirect consequence of reduced psychiatric symptoms.

In support of this, it is recognized that treatment of sleep problems may have additional benefits on mental health outcomes for patients with comorbid psychiatric illness and may prevent the onset of psychiatric conditions in ‘at-risk’ individuals. There is promising indication that treatment of insomnia through cognitive behavioural therapy for insomnia (CBT-I) can lead to improvement in the comorbid or secondary psychiatric illness such as depression [73], generalized anxiety disorder [23] and alcoholism [74]. Khurshid [75] reviews the evidence for treatment of insomnia in psychiatric settings [75].

Mindfulness has also been delivered in combination with behavioural elements of CBT-I, in a treatment called MBT-I [25, 57, 58, 76]. The conceptual basis for MBT-I is to improve sleep and daytime functioning by reducing hyperarousal [25], a prominent waking correlate that develops during the course of chronic insomnia [77]. In contrast to CBT-I that is primarily aimed at changing thoughts and behaviours to reduce unwanted wakefulness at night, MBT-I is aimed at shifting metacognitions to reduce sleep-related arousal at night and during the day through mindfulness meditation practice [25]. Ong et al. [78] found that MBT-I was marginally superior for improving subjective sleep parameters, such as total wake time and total sleep time, when compared with MBSR in individuals with chronic insomnia [78]. There is also emerging evidence that a digital MBT-I program can improve insomnia severity and pre-seep arousal in individuals with insomnia [79]. Despite the wide range of MBIs presented in the current review, we did not find any studies evaluating MBT-I for sleep in people with psychiatric disorders. There is exciting potential to investigate the usefulness of MBT-I in populations with mental illness, particularly given the observed improvements with interventions that did not directly focus on targeting sleep with mindfulness. Taken together, it is paramount that sleep is considered a critical focus of treatment in individuals with mental illness.

Given the body of research that supports the use of MBIs in both insomnia and psychiatric disorders independently, combined with results of this review and other review meta-analyses [39], there is support for using MBIs to address sleep issues in populations with comorbid psychiatric disorders. As with utilizing mindfulness meditation for insomnia or mental health in isolation, appropriate training of a mental health professional is required, as is adequate monitoring. It is not recommended to teach meditation to a client experiencing delusions or dissociation. We recommend using standardized sleep measurement to track progress, such as the ISI, alongside an appropriate measure of mental health symptoms. If deterioration occurs, treatment should be suspended.

## Conclusions

Based on the research currently available, there is promise that MBIs can be used to treat sleep disorder in those with insomnia and mental illness. However, given the lack of large-scale RCTs and heterogeneity amongst current studies in terms of treatment type, outcome measures and duration of treatment, it is premature to conclude that MBIs are effective to improve sleep for those with mental illness. We encourage the investigation of MBT-I in populations with mental illness and comorbid insomnia, given its promise as an insomnia intervention. We also suggest the inclusion of both objective and subjective sleep outcomes to further investigate the effectiveness of interventions. There is scope to strengthen our understanding of the effectiveness of MBIs for sleep in those with mental illnesses by building on existing literature with further rigorous intervention evaluation.

## Supplementary Information

Below is the link to the electronic supplementary material.

Supplementary file1 (PDF 1225 KB)

Supplementary file2 (PDF 1225 KB)

Supplementary file3 (PDF 1225 KB)

## Abbreviations

ADHD: Attention-deficit hyperactivity disorder
CBASP: Cognitive Behavioural Analysis System of Psychotherapy
CBGT: Cognitive Behavioural Group Therapy
CBT-I: Cognitive behavioural therapy for insomnia
ECG: Electrocardiogram
iMBCT: Internet MBCT
IMMI: Internet mindfulness meditation intervention
ISI: Insomnia Severity Index
MBB: Mind-body bridging
MBCT: Mindfulness-based cognitive therapy
MBI: Mindfulness-based intervention
MBSR: Mindfulness-based stress reduction
MBTT: Mindfulness-based touch therapy
MM: Mindfulness meditation
PSG: Polysomnography
PSQI: Pittsburgh Sleep Quality Index
PTSD: Post-traumatic stress disorder
RCT: Randomized control trial
SB: Slow breathing
SE: Sleep efficiency
SOL: Sleep onset latency
SWS: Slow wave sleep
TAU: Treatment as usual
TIB: Time in bed
TST: Total sleep time
TWT: Total wake time
WASO: Wake After Sleep Onset
